# Treatment of upper urinary tract stones with extracorporeal shock wave lithotripsy (ESWL) Sonolith vision

**DOI:** 10.1186/1471-2490-11-26

**Published:** 2011-12-12

**Authors:** Kogenta Nakamura, Motoi Tobiume, Masahiro Narushima, Takahiko Yoshizawa, Genya Nishikawa, Yoshiharu Kato, Remi Katsuda, Kenji Zennami, Shigeyuki Aoki, Yoshiaki Yamada, Nobuaki Honda, Makoto Sumitomo

**Affiliations:** 1 Department of Urology, Aichi Medical University School of Medicine Nagakute, Aichi 480-1195, Japan; 2 Department of Urology, Meitetsu Hospital Nagoya, Aichi 451-8511, Japan

## Abstract

**Background:**

The aim was to retrospectively assess the results of treatment of upper urinary tract stones with the Sonolith vision manufactured by EDAP, and purchased in 2004.

**Methods:**

The subjects were 226 Japanese patients who underwent extracorporeal shock wave lithotripsy (ESWL) alone as an initial treatment and could be followed up for at least 3 months, selected from 277 candidate patients who underwent this therapy between 2004 and 2006. Treatment effect was evaluated by kidney, ureter, and bladder X-ray or renal ultrasonography at 1 and 3 months after treatment. A stone-free status or status of stone fragmentation to 4 mm or smaller was considered to indicate effective treatment.

**Results:**

At 3 months after treatment, the stone-free rate was 69.4% and the efficacy rate was 77.4% for renal stones, while these rates were 91.5 and 93.3%, respectively for ureteral stones. Assessment of treatment effect classified by the location of stones revealed a stone-free rate of 94.6% and an efficacy rate of 94.6% for lower ureteral stones (4.0 mm or smaller, 1 subject; 4.1-10.0 mm, 31 subjects; 10.1-20.0 mm, 5 subjects: number of treatment sessions, 1 or 2 sessions [mean: 1.03 sessions]). Complications of this therapy included renal subcapsular hematoma and pyelonephritis in 1 case each.

**Conclusions:**

ESWL with the Sonolith vision manufactured by EDAP produced a treatment effect equivalent to those achieved with other models of ESWL equipment. ESWL seems to be an effective first-line treatment also in patients who have lower ureteral stones 10 mm or larger but do not wish to undergo TUL, if measures such as suitable positioning of the patient during treatment are taken.

## Background

Extracorporeal shock wave lithotripsy (ESWL) was introduced in clinical practice by Chaussy *et al. *in the 1980 s [1]. Its usefulness is widely recognized, and it has become the most common treatment for upper urinary tract stones. ESWL has been performed more frequently than TUL for stone treatment in Japan compared with that in Europe and the United States (2008 General Meeting, Seminar of the Japanese Urological Association), which may be largely attributable to its simplicity and/or the National Health Insurance System in Japan. Our hospital introduced the Sonolith vision (manufactured by EDAP) in March 2004 in place of the old equipment, and has been performing ESWL on patients with upper urinary tract stones. Here we report the favorable results of treatment of lower ureteral stones in cases where lithotripsy was difficult, which were achieved by putting the patient in a suitable position.

## Methods

The present study was reviewed and approved by the Ethics Committee of Aichi Medical University School of Medicine (No. 11-015). The subjects were 226 Japanese patients who underwent ESWL alone and could be followed up for at least 3 months, selected from 277 candidate patients who underwent this therapy as the initial treatment between March 1, 2004 and December 31, 2006. The subjects included 169 male and 57 female aged from 17 to 86 years (mean: 50.5 years). The stones were on the left side in 128 subjects and on the right side in 98. The locations and sizes of the stones are shown in Table 1.

**Table 1:** Location and size of stones

	≤ 4.0 mm	4.1-10.0 mm	10.1-20.0 mm	≥ 20.1 mm	Total (n)
Middle renal		5	36	11	52
					
Lower renal		2	7	1	10
					
Upper ureteral		77	35	1	113
					
Middle ureteral		11	3		14
					
Lower ureteral	1	31	5		37

Total (n)	1	126	86	13	226

Treatment was performed during a hospital stay of three days and two nights, and the subjects were only fasted before operation. Preoperative medication included diclofenac suppository, Atarax-P, atropine sulfate (i.m.), and glycerin enema (60 mL). When subjects complained of severe pain during operation, pentazocine (i.m.) was additionally administered. Renal stones were treated at a maximum energy level of 80%, while ureteral stones were treated at a level of 100%. Both renal and ureteral stones were treated with up to 3000 shock waves. Treatment effect was evaluated by kidney, ureter, and bladder (KUB) X-ray or intravenous pyelography at 1 and 3 months after operation, and was classified as stone-free status (absence of residual stones), effective (presence of residual stones 4 mm or smaller), inadequate (status other than the above), or ineffective (no stone fragmentation even after 2 treatment sessions of ESWL).

**Figure 1:**
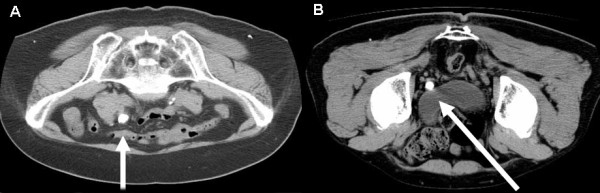
**A) ESWL approaches to middle ureteral stones**. (Ipsilateral prone position). B) ESWL approaches to lower ureteral stones. (Contralateral prone position)

Treatment was completed if an effective or better response was demonstrated by KUB on the day after the operation. If stone fragmentation was found to be inadequate, a second ESWL session was performed after about 1 to 4 weeks. If any stone fragmentation was achieved, third and subsequent ESWL sessions were performed. Subjects with an inadequate or no response (ineffective) to ESWL underwent transurethral ureterolithotripsy (TUL) or percutaneous nephrolithotripsy (PNL). Subjects with urinary tract infection or with stones 20 mm or larger underwent placement of a double-J stent. During the procedure, subjects with renal or upper ureteral stones were placed in the ipsilateral supine position, those with middle ureteral stones in the ipsilateral prone position, and those with lower ureteral stones in the contralateral prone position (Figure 1). Subjects with X-ray-negative stones underwent intravenous pyelography (IVP) and ureteral catheter insertion in combination with ESWL.

## Results

Of the 226 cases, 30 cases underwent placement of a ureteral stent and 14 cases underwent IVP. When the stones were classified by location, middle renal stones were the most frequent (52 cases) among subjects with renal stones, while upper ureteral stones (113 cases) were the most frequent among subjects with ureteral stones. When the stones were classified by size, those 10.1 - 20.0 mm were the most frequent (43 cases) of renal stones, while those 4.1 - 10.0 mm were the most frequent (119 cases) of ureteral stones. One to 10 treatment sessions (mean: 1.62 sessions) were performed. Renal stones were treated with a mean of 1.8 sessions, of which middle renal stones of 20.1 mm or larger were treated with the largest number of sessions (mean: 3.9 sessions). Ureteral stones were treated with a mean of 1.44 sessions, of which middle ureteral stones of 10.1 - 20.0 mm or larger were treated with the largest number of sessions (mean: 3.6 sessions) (Table 2). One month after operation, the stone-free rate was 48.4% and the efficacy rate was 64.5% for renal stones, while these rates were 70.7 and 80.5%, respectively for ureteral stones. Three months after operation, the stone-free rate was 69.4% and the efficacy rate was 77.4% for renal stones, while these rates were 91.5 and 93.3%, respectively for ureteral stones. When the treatment effect was analyzed by the location of stones, a stone-free rate of 94.6% and an efficacy rate of 94.6% were achieved in subjects with lower ureteral stones (≤ 4.0 mm, 1 subject; 4.1 - 10.0 mm, 31 subjects; 10.1 - 20.0 mm, 5 subjects) with one or two ESWL treatment sessions (mean: 1.03 sessions) at three months after operation (Table 3). All five subjects with stones 10 mm or larger achieved a complete stone-free status.

**Table 2:** Location and size stones and number of treatment

		Number of treatment sessions						Mean (session)
		1	2	3	4	6	10	
	4.1-10.0 mm	4	1					1.2
Middle renal	10.1-20.0 mm	20	9	3	4			1.8
	≥ 20.1 mm	1	1	4	3	1	1	3.9
								
	4.1-10.0 mm	2						1
Lower renal	10.1-20.0 mm	3	3			1		2.1
	≥ 20.1 mm	1						1

	4.1-10.0 mm	66	9	2				1.2
Upper ureteral	10.1-20.0 mm	28	5	2				1.3
	≥ 20.1 mm	1						1
								
	4.1-10.0 mm	10		1				1.2
Middle ureteral	10.1-20.0 mm		1	1		1		3.6
								
	4.1-10.0 mm	1						1
Lower ureteral	10.1-20.0 mm	31						1
	≥ 20.1 mm	4	1					1.2

Total(n)		172	30	13	7	3	1	1.62

**Table 3:** Treatment results by location

		1 month		3 months	
**Site**	**No of pts**	**Stone-free rate**	**Efficacy rate**	**Stone-free rate**	**Efficacy rate**

Middle renal	52	53.80%	69.20%	71.20%	78.80%
Lower renal	10	20.00%	40.00%	60.00%	70.00%
					
Total (renal)	62	48.40%	64.50%	69.40%	77.40%

Upper ureteral	113	73.50%	83.20%	89.40%	92.00%
Middle ureteral	14	64.30%	71.40%	100%	100%
Lower ureteral	37	64.90%	75.70%	94.60%	94.60%
					
Total (ureteral)	164	70.70%	80.50%	91.50%	93.30%

Fourteen subjects with X-ray-negative stones underwent intravenous pyelography (IVP) and ureteral catheter insertion in combination with ESWL. These patients had a stone-free status with only ESWL therapy.

Of the 25 cases in whom ESWL was ineffective, 8 cases were confirmed to be stone free three or more months after operation, while 7 cases with residual stones were followed up because hydronephrosis improved. Of the subjects with upper ureteral stones in whom ESWL was ineffective, 7 cases underwent TUL and 2 cases underwent PNL. One case with concomitant ureteral stenosis underwent holmium laser incision following stone fragmentation with TUL.

The chemical composition of stones could be determined in 128 cases. Calcium oxalate stones were the most frequent (68 cases), followed by mixed calcium oxalate and calcium phosphate stones (49 cases), and stones containing calcium were present in 126 cases.

All subjects experienced postoperative gross hematuria as a complication. One case developed renal subcapsular hematoma which improved with conservative treatment. One case developed fever of 38°C or higher, which resolved with antibiotic therapy. Fifteen subjects required pentazocine for pain during the operative procedure.

## Discussion

Since Chaussy *et al. *[1] reported the application of ESWL with an HM-3 lithotriptor manufactured by Dornier Co., Ltd. in clinical practice in the 1980 s, various models of ESWL equipment have been developed and improved. Shock wave generators have been developed, starting with the underwater spark gap type, followed by the electromagnetic conversion type and the piezoelectric element type. ESWL is now the first-line treatment for upper urinary tract stones.

Our hospital introduced the Sonolith vision (manufactured by EDAP) on March 1, 2004. This model belongs to third generation ESWL equipment. It uses electrical conduction electrodes as the shock wave generator, which generates shock waves in a highly electrical-conductive fluid. It provides accurate electrical discharge with high reproducibility, thus producing stable and constant energy. The use of a hydrophone pressure detector allows real-time display of the effective pressure of shock waves on a monitor. The generator is a shallow oval shape, and the output power can be adjusted to 100 different levels. The diameter of the shock-wave head is as large as 22 cm, and the consumptive electrodes can be used in four to five patients. This equipment has a focal depth of 130 mm and a focal size of 3 × 28 mm. The focal point is adjusted using an X-ray C-arm.

In 226 Japanese cases with renal or ureteral stones who were treated with ESWL in our hospital, the stone-free rate was 85.4% and the efficacy rate was 88.9%, showing similar results to those obtained by other researchers [2-5]. (Table 4).

**Table 4:** Treatment results by model

	**White *et al***.	**Johnson *et al***.	Egilmez *et al*	**Nomikos *et al***.	Our institute
**Manufacturer**	**Dornier**	**Dornier**	**Siemens**	**EDAP**	**EDAP**
					
Model	Delta lithotriptor	Delta S lithotriptor	Lithostar	Sonolith vision	Sonolith vision
					
No. of cases	5735	270	2670	309	226
					
Location of Stone	kidney/ureter	kidney/ureter	kidney/ureter	kidney	kidney/ureter
					
Stone-free rate (%)	58.5	73.3	79	75	85.4
					
Eficacy rate (%)	85.1	85.6			88.9

With regard to lower ureteral stones, Hochreiter *et al. *[6] treated distal ureteral stones with the HM-3 lithotriptor manufactured by Dornier in 518 patients, and reported a stone-free rate of 97.3% and efficacy rate of 99.4%. Park *et al. *[7], however, reported that the stone-free rate was 55.6% for lower ureteral stones of 10 mm or larger. Pardalidis *et al. *[8] also reported that treatment was not so effective, with a stone-free rate of 84.6%. Ghalayini *et al. *[10] compared laser TUL with ESWL, and reported that laser TUL was significantly more effective than ESWL, with a stone-free rate of 97.5% vs. 81.5% three months after operation. Wu *et al. *[11] compared holmium yttrium-aluminum-garnet (YAG) laser TUL with ESWL in patients with upper ureteral stones, and reported that there was no significant difference in the treatment effect on stones 10 mm or smaller, with a stone-free rate of 91.1% for TUL vs. 85.3% for ESWL, while there was a significant difference in the treatment effect on stones 10 mm or larger, with a stone-free rate of 76.8% for TUL vs. 35.2% for ESWL. They stated that laser TUL was superior to ESWL for the treatment of stones 10 mm or larger. In our study, however, all subjects with stones 10 mm or larger achieved a stone-free status. This may be attributable to our new measures: we usually place patients in the contralateral prone position to potentiate the treatment effect on lower ureteral stones, in accordance with the report by Köse *et al. *[12], and before ultrasonography we also apply jelly in a thin layer to the skin surface and to the area on the treatment table around the spot in contact with the skin to avoid exposure to air, thereby reducing minute air bubbles generated from the skin surface and minimizing attenuation of shock waves. ESWL seems to be an effective first-line treatment also in subjects who have lower ureteral stones 10 mm or larger but do not wish to undergo TUL, but it is sometimes difficult to treat stones greater than 10 mm by ESWL monotherapy. So, it is necessary to perform ESWL and TUL combination therapy. In contrast, these subjects with X-ray-negative stones underwent intravenous pyelography (IVP) and ureteral catheter insertion in combination with ESWL. These patients of ours had a stone-free status with only ESWL therapy.

Madbouly *et al. *[13] recently reported that the treatment effect of ESWL with the Lithostar Multiline performed under general anesthesia was significantly greater when shock waves were delivered at a slow rate (60/min) than at a rapid rate (120/min), with an efficacy rate of 98.7% vs. 90.0%. Pace *et al. *[14] and Peterson *et al. *[15] also reported similar results. Thus, ESWL treatment should be performed at a slow rate in the future.

Complications of ESWL include renal subcapsular hematoma, the incidence of which has been reported to be between 0.078% and 0.6% [1,16], and has also been reported to increase up to 32% as a result of performance of postoperative routine CT and MRI [17]. Risk factors for renal subcapsular hematoma are reported to be 1) hypertension, 2) coagulopathy, and 3) previous ESWL therapy [18]. In our study, renal subcapsular hematoma occurred in one subject. This subject had a history of hypertension, which may have caused this complication.

## Conclusions

ESWL with the Sonolith vision manufactured by EDAP produced a treatment effect equivalent to those achieved with other models of ESWL equipment. ESWL seems to be an effective first-line treatment also in patients who have lower ureteral stones 10 mm or larger but do not wish to undergo TUL, if measures such as suitable positioning of patients during treatment are taken.

## Competing interests

The authors declare that they have no competing interests.

## Authors' contributions

KN and MT drafted the report, cared for the patient and approved the final version of the manuscript. MN, TY, GN, YK, RK, KZ, SA, YY and NH cared for the patient and approved the final version of the manuscript. MS approved the final version of the manuscript.

## Pre-publication history

The pre-publication history for this paper can be accessed here:

http://www.biomedcentral.com/1471-2490/11/26/prepub
